# Unilateral upper lung field pulmonary fibrosis after primary lung cancer surgery as a late complication to be recognized

**DOI:** 10.1007/s11748-025-02164-9

**Published:** 2025-06-06

**Authors:** Hironori Ishibashi, Mariko Hanafusa, Ayaka Asakawa, Yuya Ishikawa, Ryo Wakejima, Shota Horibe, Kenichi Okubo

**Affiliations:** 1 Department of Thoracic Surgery, Graduate School of Medical and Dental Sciences, Institute of Science Tokyo, 1-5-45 Yushima, Bunkyo-ku, Tokyo, 113-8519 Japan; 2 Division of Cohort Research, National Cancer Center Institute for Cancer Control, 5-1-1 Tsukiji, Chuo-ku, Tokyo, 104-0045 Japan

**Keywords:** Lung cancer, Thoracic surgery, Upper lung field pulmonary fibrosis

## Abstract

**Objective:**

Unilateral upper lung field pulmonary fibrosis (UPF) is a possible complication on the operated side after lung cancer surgery. However, its incidence and associated perioperative factors remain unclear. This study investigated the clinical characteristics of patients with unilateral UPF after primary lung cancer surgery.

**Methods:**

We reviewed the records of all consecutive patients with lung cancer who underwent complete resection at the Institute of Science, Tokyo, between July 2010 and December 2021. We estimated the cumulative incidence and sub-hazard ratios using competing risk regression models.

**Results:**

A total of 979 patients were included in this analysis. The median follow-up period up to the last follow-up was 59.2 months (interquartile range 37.0–84.6 months). With 39 (4.0%) cases of postoperative unilateral UPF, the median follow-up time until the diagnosis of unilateral UPF was 25.5 months (interquartile range 12.9–45.3 months), and the 3-, 5-, and 10-year cumulative incidences of unilateral UPF were 2.7%, 4.0%, and 5.4%, respectively. The 5-year overall survival rate was 87.3%; however, 30 of the 39 patients (76.9%) with unilateral UPF experienced subsequent complications related to unilateral UPF, such as progressive respiratory distress, progressive body weight loss, and pneumonia. Age > 75 years, male sex, low body mass index (< 20 kg/m^2^), ischemic heart disease, history of pneumonia, emphysema, pulmonary apical cap, and right lower lobe tumors are possible risk factors for unilateral UPF.

**Conclusions:**

Unilateral UPF is an unrecognized late complication of lung cancer surgery that should be carefully monitored in patients with risk factors.

## Introduction

Pleural parenchymal fibroelastosis (PPFE), first reported by Frankel et al. in 2004, is a rare idiopathic interstitial pneumonia characterized by prominent pleural and parenchymal infiltration, mainly in the bilateral upper lobes, and is more likely to develop in patients with a flattened chest [1–3]. PPFE often presents with restrictive pulmonary dysfunction and has a poor prognosis, with a median overall survival of 35.3 months and 1-, 3-, and 5-year survival rates of 88.5%, 45.5%, and 28.9%, respectively [4]. On the other hand, it has long been known among thoracic surgeons that progressive pleural thickening and pulmonary parenchymal fibrosis similar to PPFE occur in the unilateral upper lung field during follow-up after lung resection, but this has rarely been discussed because the cause and treatment are unknown.

Recently, unilateral upper lung field pulmonary fibrosis (UPF), radiologically consistent with PPFE, has been reported to develop as a late complication after thoracic surgery [5–8]. Postoperative unilateral UPF lesions were limited to the operated side but were similar to PPFE in terms of radiological and clinical characteristics. Unilateral UPF occurs several years after surgery and is considered an important late complication of thoracic surgery. The prognosis is reported to be poor, with a median survival time of 49.3 months, and all causes of death are respiratory diseases [6]. However, only a limited number of reports have been published, and little is known about the incidence, mechanisms, and perioperative factors associated with the development of unilateral UPF after lung cancer surgery.

This study aimed to clarify the cumulative incidence of unilateral UPF in patients who underwent complete resection of lung cancer at a single center using a competing risk model, clinical course, and prognosis of patients who developed unilateral UPF. The study also compared the background and tumor factors of patients with and without unilateral UPF and comprehensively examined possible risk factors, including surgical procedures. Finally, we describe the surgical techniques and postoperative complications in the subgroups of patients with and without unilateral UPF who underwent lobectomy.

## Materials and methods

### Patients

**Fig. 1:**
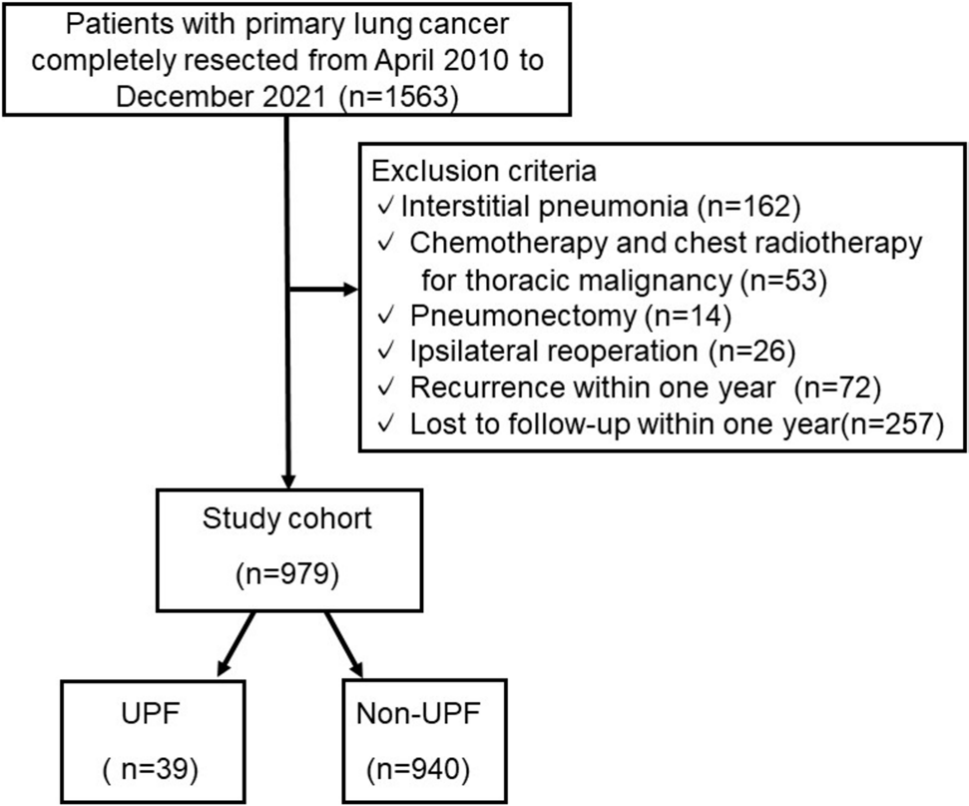
Patients’ flowchart

Data were collected from our database and patients’ electronic medical records, which included information on perioperative patient characteristics (age, sex, Eastern Cooperative Oncology Group performance status, BMI, comorbidity, smoking status, presence of apical cap, percentage vital capacity [%VC], percentage of forced expiratory volume in one second [FEV1.0%]), tumor characteristics (histological type, pathological staging, tumor site), surgical factors (previous contralateral operation, surgical procedure), and postoperative course. We reviewed the medical records of patients who underwent surgery for primary non-small cell lung cancer between April 2010 and December 2021 at the Institute of Science, Tokyo, Japan. The exclusion criteria were interstitial pneumonia, perioperative chemotherapy or radiotherapy, chest radiotherapy for a previous thoracic malignancy, pneumonectomy, ipsilateral reoperation for thoracic surgery, recurrence within 1 year, and loss to follow-up within 1 year, including death (Fig. 1).

### Ethics statement

This study was approved by the Institutional Review Board of the Institute of Science, Tokyo Hospital (certificate number M2017-326). The records of all eligible patients were reviewed using our prospectively collected database and confirmed by attending physicians.

### Anesthesia, surgical approach, and intraoperative procedures

For video-assisted thoracic surgery (VATS) lobectomy, a 2–4 cm access thoracotomy was placed in the 4 th or 5 th intercostal space in the anterior axillary line, and two 1-cm incisions were made in the 7 th intercostal space in the anterior and posterior axillary lines. Open thoracotomy was performed using a 10–15 cm anterolateral or posterolateral incision through the 4 th or 5 th intercostal space. The procedure for the pulmonary vessels and bronchi was performed using the same technique as that used for VATS and open thoracotomy. Lymph node dissection was classified as ND0 (no lymph node dissection), ND1 (lymph node dissection up to the ipsilateral hilar lymph nodes), or ND2 (lymph node dissection up to the ipsilateral mediastinal lymph nodes or above). Pathological lung cancer staging was performed according to the 8 th edition of the TNM classification of malignant tumors [9]. In each case, attempts were made to minimize leakage using additional sutures, sealants, or staple line buttressing. Five milliliters of fibrin glue (Bolheal: Teijin, Tokyo, Japan, or Beriplast P Combi-Set; CSL Behring, Tokyo, Japan) and/or polyglycolic acid (PGA) sheets were used to minimize air leakage. The chest tube removal criteria were as follows: no visually detectable air leak, correctly re-expanded lungs on chest radiography, and chest drainage fluid volume of < 200 mL within 24 h. Autologous blood patches, OK-432 (Picibanil), or talc were routinely used in patients with persistent air leaks (> 5 days) after obtaining informed consent.

### Parameters

Thin-slice high-resolution computed tomography (CT) (1 mm) was routinely performed before lung cancer surgery in all eligible patients. UPF was diagnosed when radiological findings obtained with CT matched the criteria of “definite UPF” or “consistent with UPF” previously reported as follows: “Definite UPF” was defined as demonstrating pleural thickening associated with subpleural fibrosis concentrated in the upper lobes, with markedly less or absent involvement of the lower lobes. “Consistent with UPF” was defined as pleural thickening associated with subpleural fibrosis in the upper lobes, but the distribution of these changes was not concentrated in the upper lobes, nor were there features of coexistent disease elsewhere [10]. These criteria were applied only to unilateral lungs on the operated side. The diagnosis of UPF was diagnosed based on the consensus of at least two board-certified chest surgeons and radiologists. A pulmonary apical cap is a wedge- and triangle-shaped opacity in the apex of the lung with broad pleural contact [11], which was diagnosed using CT by two board-certified thoracic surgeons. During the postoperative follow-up period, chest radiography was performed every six months for the first five years, after which CT was performed every 12 months, typically for up to 10 years.

The following variables were categorized as follows: age (< 75 or ≥ 75 years) [12], BMI (< 18.5, 18.5–22.9, or 23 + kg/m^2^) according to the Asian-specific BMI cutoff value [13], lobe (left upper and lower lobe (Left), right upper lobe (RU), right lower lobe (RL), middle lobe/multi lobes (others)), and postoperative chest drainage duration (≤ 4 days or > 5 days) [14].

Postoperative complications that developed within 30 days after surgery were defined according to the Clavien–Dindo classification as grade IIIa or higher [15]; those not defined by the Clavien–Dindo classification were defined according to the Common Terminology Criteria for Adverse Events (version 5.0) as grade III. Postoperative pleural effusion was defined as pleural effusion requiring re-drainage during hospitalization or after discharge. Postoperative hematoma was defined as a hematoma requiring reoperation or drainage. Postoperative pleuritis was defined as the requirement for antibiotics for more than one week because of persistent inflammatory findings without pneumonia or wound infection.

### Statistical analysis

Continuous variables are presented as the mean (± standard deviation: SD) or median (interquartile range (IQR)), and differences between groups were evaluated using t-tests or the Wilcoxon rank-sum test. Categorical variables are presented as numbers (%), and differences between groups were evaluated using the chi-squared (*χ*^*2*^) test. Statistical significance was set at *p* < 0.05. The competing risk method was used to estimate the cumulative incidence of unilateral UPF. In this study, the treatment of metachronous ipsilateral multiple lung cancers or death before the onset of unilateral UPF was considered a competing risk. Patients who did not develop unilateral UPF or competing risks were censored at the last follow-up or 10 years postoperatively, whichever occurred first. For the entire population, a minimally adjusted (age and sex) proportional hazards model (Fine–Gray model) was used to determine the factors associated with the incidence of unilateral UPF among patients and tumor characteristics. As there were two missing values for %VC and FEV1.0%, multiple imputations by chained equations with 20 imputed datasets were used to analyze these variables [16].

Statistical analyses were performed using Stata software (version 18.0; Stata Corp LP, College Station, TX, USA), and graphs of cumulative incidence were generated using R software (The R Foundation for Statistical Computing, Vienna, Austria) [17].

## Results

### Patient background

Between April 2010 and December 2021, 1563 cases of completely resected primary lung cancers were reported. Of the 162 patients with interstitial pneumonia, 53 who received chemotherapy and/or chest radiotherapy for thoracic malignancy, 14 who underwent pneumonectomy, 26 who underwent ipsilateral reoperation, 72 who relapsed within one year after surgery, and 257 who could not be followed up for more than one year after surgery were excluded (Fig. 1). A total of 979 patients were included in this analysis. The median follow-up period up to the last follow-up was 59.2 months (IQR 37.0–84.6 months). Unilateral UPF was observed in 39 (4.0%) of the 979 patients.

The patient demographics are summarized in Table 1. There were 576 males (58.8%) and 403 (41.2%) females, with a mean (SD) age of 69.3 (± 9.1), ranging from 29 to 89 years. Patients in the UPF group were older, comprised a larger proportion of men, and had a lower BMI. The incidence of ischemic heart disease, history of pneumonia and emphysema, and the presence of an apical cap were significantly higher in the UPF group.

**Table 1:** Patient and tumor characteristics by upper lung field pulmonary fibrosis status. (n = 979)

		Total	UPF	non-UPF	*P*-Value
		(*n* = 979)	(*n* = 39)	(*n* = 940)	
*Patient characteristics*					
Age (years), n (%)	< 75	680 (69.5%)	22 (56.4%)	658 (70.0%)	0.071
	75 +	299 (30.5%)	17 (43.6%)	282 (30.0%)	
Sex, n (%)	Male	576 (58.8%)	29 (74.4%)	547 (58.2%)	*0.044*
	Female	403 (41.2%)	10 (25.6%)	393 (41.8%)	
ECOG performance status, n (%)	0	900 (91.9%)	37 (94.9%)	863 (91.8%)	0.49
	1 +	79 (8.1%)	2 (5.1%)	77 (8.2%)	
Body mass index (kg/m2), n (%)	< 18.5	71 (7.3%)	7 (17.9%)	64 (6.8%)	*0.016*
	18.5–22.9	442 (45.1%)	19 (48.7%)	423 (45.0%)	
	23 +	466 (47.6%)	13 (33.3%)	453 (48.2%)	
*Cardiovascular comorbidity*					
Hypertension, n (%)		420 (42.9%)	18 (46.2%)	402 (42.8%)	0.68
Ischemic heart disease, n (%)		80 (8.2%)	8 (20.5%)	72 (7.7%)	*0.004*
Cerebral infarction, n (%)		90 (9.2%)	4 (10.3%)	86 (9.1%)	0.81
*Respiratory comorbidity*					
Past history of pneumonia, n (%)		69 (7.0%)	7 (17.9%)	62 (6.6%)	*0.007*
Preoperative atelectasis, n (%)		14 (1.4%)	1 (2.6%)	13 (1.4%)	0.54
Past history of tuberculosis, n (%)		63 (6.4%)	2 (5.1%)	61 (6.5%)	0.73
Asthma, n (%)		53 (5.4%)	2 (5.1%)	51 (5.4%)	0.94
Emphysema, n (%)		417 (42.6%)	25 (64.1%)	392 (41.7%)	*0.006*
*Others*					
Diabetes mellitus, n (%)		177 (18.1%)	8 (20.5%)	169 (18.0%)	0.69
Chronic renal failure, n (%)		24 (2.5%)	1 (2.6%)	23 (2.4%)	0.96
Previous cancer, n (%)		270 (27.6%)	12 (30.8%)	258 (27.4%)	0.65
Anticoagulant agents, n (%)		200 (20.4%)	11 (28.2%)	189 (20.1%)	0.22
Smoking status, n (%)	Never	302 (30.8%)	8 (20.5%)	294 (31.3%)	0.33
	Past	458 (46.8%)	22 (56.4%)	436 (46.4%)	
	Current(< 12 M)	219 (22.4%)	9 (23.1%)	210 (22.3%)	
Apical cap, n (%)		443 (45.3%)	34 (87.2%)	409 (43.5%)	< *0.001*
%VC, mean (SD)		107.7 (15.9)	106.4 (16.7)	107.7 (15.8)	0.60
FEV 1.0%, mean (SD)		92.4 (18.6)	91.9 (19.1)	92.4 (18.6)	0.89
*Tumor characteristics*					
Histological type, n (%)	Adeno- carcinoma	765 (78.1%)	27 (69.2%)	738 (78.5%)	0.20
	Squamous carcinoma	152 (15.5%)	10 (25.6%)	142 (15.1%)	
	Others	62 (6.3%)	2 (5.1%)	60 (6.4%)	
Pathological stage, n (%)	I	744 (76.0%)	25 (64.1%)	719 (76.5%)	0.21
	II	131 (13.4%)	8 (20.5%)	123 (13.1%)	
	III	104 (10.6%)	6 (15.4%)	98 (10.4%)	
Side, n (%)	Left	376 (38.4%)	11 (28.2%)	365 (38.8%)	0.18
	Right	603 (61.6%)	28 (71.8%)	575 (61.2%)	
Surgical procedure, n (%)	Partial	35 (3.6)	1 (2.6)	34 (3.6)	0.39
	Segmentectomy	146 (14.9)	3 (7.7)	143 (15.2)	
	Lobectomy	798 (81.5)	35 (89.7)	763 (81.2)	
Lobe, n (%)	Left	376 (38.4%)	11 (28.2%)	365 (38.8%)	< *0.001*
	Right upper	345 (35.2%)	5 (12.8%)	340 (36.2%)	
	Right lower	193 (19.7%)	22 (56.4%)	171 (18.2%)	
	Other	65 (6.6%)	1 (2.6%)	64 (6.8%)	

*ECOG* Eastern cooperative oncology group, *FEV 1.0%* forced expiratory volume in 1 s%, *%VC* = % vital capacity, *SD* standard definition, *UPF* upper lung field pulmonary fibrosis

### Cumulative incidence

**Fig. 2:**
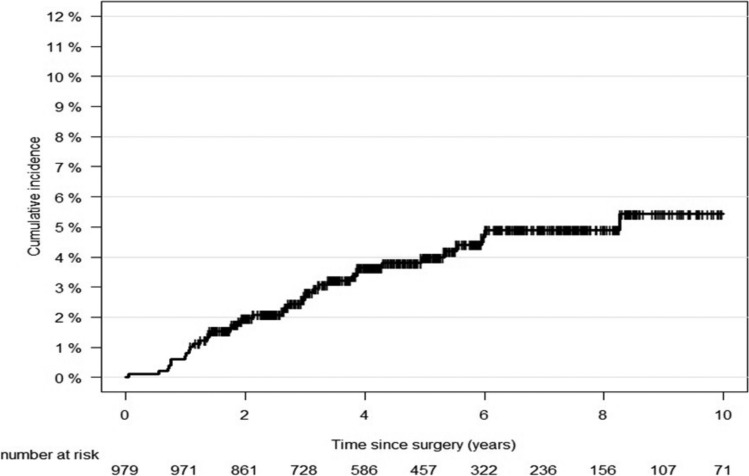
Cumulative incidence curves of unilateral UPF after lung cancer surgery. (*n* = 979)

The cumulative incidence of unilateral UPF gradually increased after lung cancer surgery (Fig. 2). In the total population, the 1-, 3-, 5-, and 10-year cumulative incidences of unilateral UPF were 0.6%, 2.7%, 4.0%, and 5.4%, respectively.

### Clinical and radiological courses

**Fig. 3:**
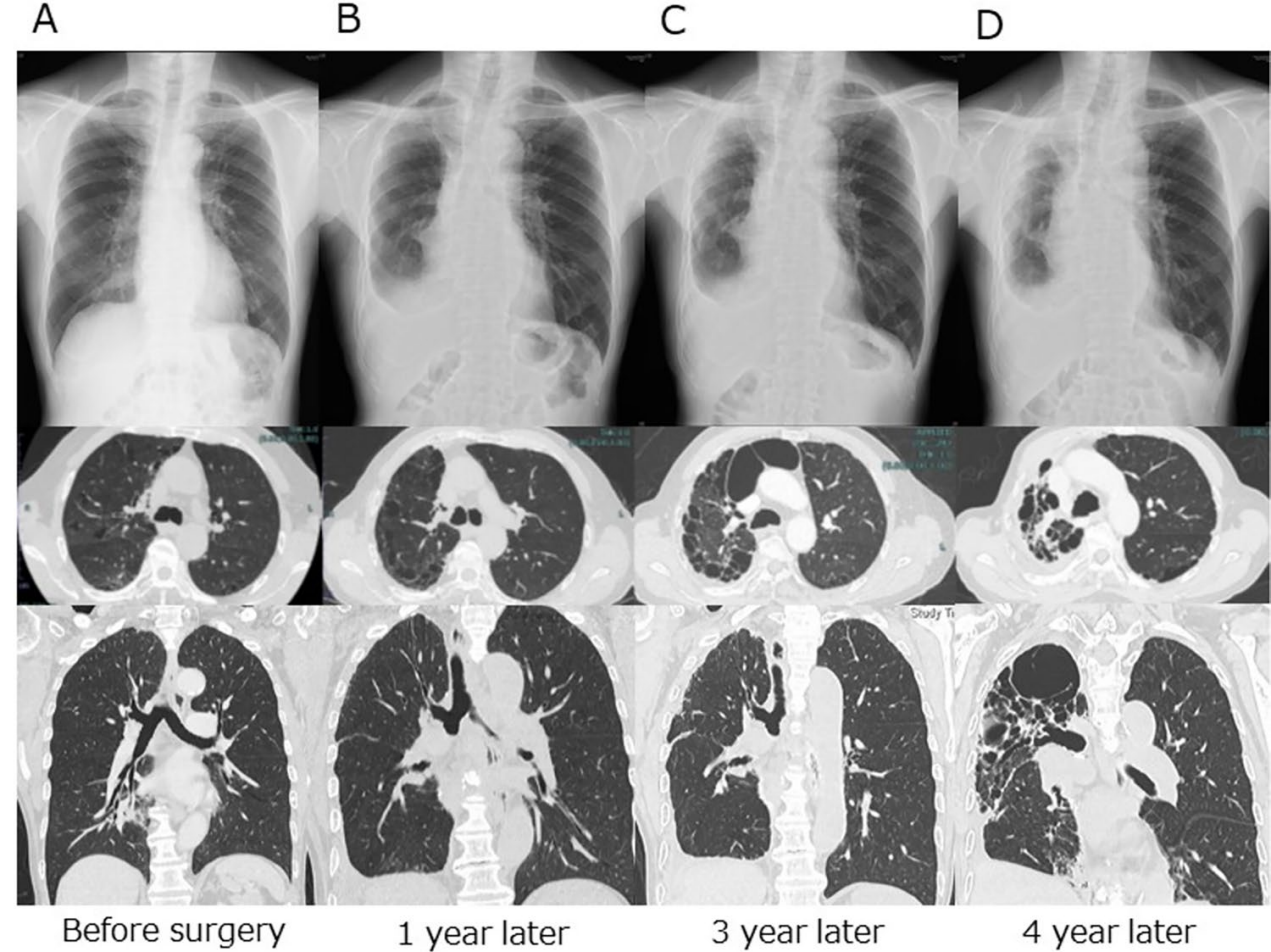
Radiological course of a 74-year-old male patient who underwent right lower lobectomy for stage IA1 adenocarcinoma via VATS. **A** Before surgery, there is no mediastinal shift. **B** Postoperative chest X-ray and computed tomography one year after surgery showing right pleural effusion. **C** Three years after surgery, a subpleural parenchymal lesion in the right upper lobe showed slight fibrosis with pleural spicula or indentation and a mediastinal shift. **D** Four years after surgery, the cystic lesion in the right upper lung field is obviously deteriorated with unilateral thoracic deformity

**Fig. 4:**
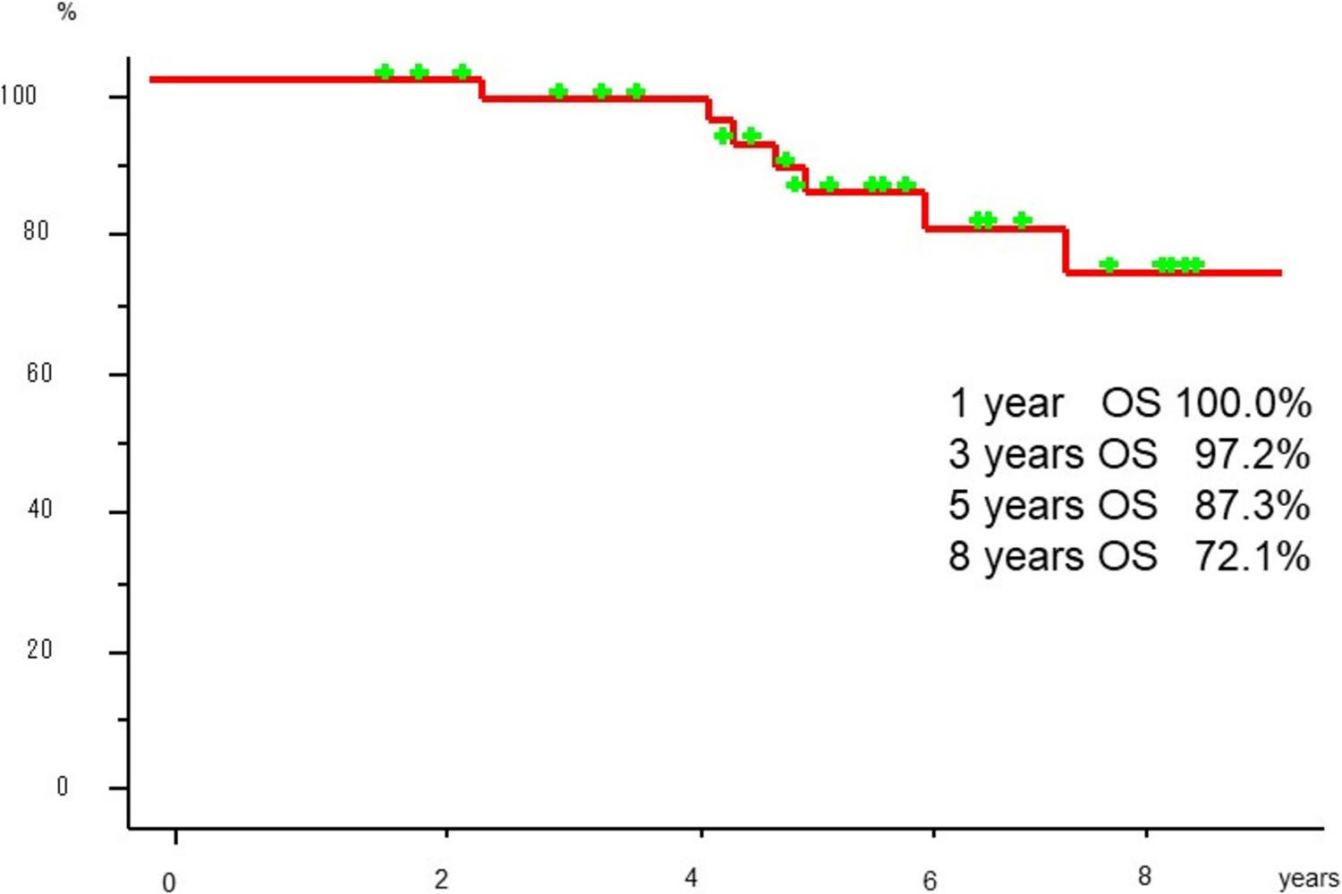
The 1-, 3-, 5-, and 8-year overall survival rates after being diagnosed with unilateral UPF are 100.0%, 97.2%, 87.3%, and 72.1%, respectively

The clinical and radiological characteristics of the patients with unilateral UPF are shown in Table 2. The median interval from lung cancer surgery to the diagnosis of unilateral UPF was 25.5 months (IQR 12.9–45.3 months). During the radiological course, the unilateral UPF lesions initially showed slight fibrosis with pleural spicula, indentation, and shift of the mediastinum, and the size of the thoracic cage on the operated side gradually decreased in all patients (Fig. 3). Thirty patients (76.9%) experienced adverse events caused by unilateral UPF, including progressive respiratory distress in 18 patients (46.2%), worsening of ECOG performance status in 10 patients (25.6%), progressive body weight loss of 5% or more of total body weight within 6 months in 12 patients (30.8%), and pneumonia in five patients (12.8%) during the observation period. Seven patients died of lung cancer; however, none died of UPF-related causes during follow-up. The pathological background of lung specimens resected for lung cancer in 39 patients with unilateral UPF was as follows: normal in five patients, inflammatory changes in two patients, emphysematous changes in seven patients, emphysema with inflammatory changes in 15 patients, and interstitial pneumonia in 10 patients without radiological evidence of an interstitial shadow on preoperative CT. The 1-, 3-, 5-, and 8-year overall survival rates after the diagnosis of unilateral UPF were 100.0%, 97.2%, 87.3%, and 72.1%, respectively (Fig. 4).

**Table 2:** The clinical and radiological courses of the 39 patients with UPF

Clinical courses	
Median interval time from surgery to diagnosis of UPF (range)	25.5 months (12.9–45.3)
Subsequent complication related to UPF, n (%)	
Present	30 (76.9)
Progressive respiratory distress	18
Worsening of ECOG performance status	10
Progressive body weight loss of 5% or more of total body weight within 6 months	12
Pneumonia	5
Absent	9 (23.1)
Unilateral thoracic deformity, n (%)	
Present	39 (100.0)
Absent	0 (0.0)
Prognosis, n (%)	
Alive	29 (74.4)
Dead	10 (25.6)
Causes of death, n	
Recurrent lung cancer	7
Liver cirrhosis	1
Aortic dissection	1
Unknown	1
Pathological finding of resected lung, n (%)	
Normal	5 (12.8)
Interstitial pneumonia	10 (25.6)
Emphysematous lung	7 (17.9)
Inflammatory change	2 (5.1)
Emphysematous lung with inflammatory change	15 (38.6)

*UPF*: upper lung field pulmonary fibrosis

### Risk factor analysis

The minimally adjusted analyses of the unilateral UPF are presented in Table 3. Age > 75 years, female sex, presence of ischemic heart disease, history of pneumonia, presence of emphysema, presence of an apical cap, and RL tumors were significantly associated with the incidence of unilateral UPF.

**Table 3:** The results of minimally adjusted proportional hazards model

		SHR	95% CI		P-value
*Patient characteristics*					
Age (years)	< 75	Ref			
	75 +	2.01	1.06	3.81	**0.031**
Sex	Male	Ref			
	Female	0.48	0.24	0.997	**0.049**
ECOG performance status	0	Ref			
	1 +	0.46	0.10	1.99	0.296
Body mass index (kg/m2)	< 20	2.69	1.11	6.50	**0.028**
	20–24	Ref			
	25 +	0.59	0.29	1.19	0.140
*Comorbidities (Ref. No)*					
Hypertension		1.04	0.54	2.00	0.899
Ischemic heart disease		2.36	1.04	5.34	**0.039**
Cerebral infarction		1.01	0.35	2.88	0.989
Past history of pneumonia		2.74	1.26	5.96	**0.011**
Preoperative atelectasis		1.77	0.25	12.46	0.565
Past history of tuberculosis		0.69	0.17	2.75	0.597
Asthma		1.02	0.23	4.45	0.980
Emphysema		2.15	1.09	4.24	**0.027**
Diabetes mellitus		1.01	0.46	2.22	0.982
Chronic renal failure		0.88	0.11	6.94	0.902
Previous cancer		1.20	0.61	2.36	0.599
Anticoagulant agents		1.33	0.66	2.66	0.427
*Others*					
Smoking status	Never	Ref			
	Past	1.40	0.55	3.56	0.484
	Current(< 12 M)	1.27	0.40	4.03	0.685
Apical cap (Ref. No)		8.40	3.24	21.77	** < 0.0001**
%VC (mL)	(per one unit increase)	0.997	0.98	1.02	0.775
FEV 1.0% (mL)	(per one unit increase)	1.01	0.99	1.02	0.480
*Tumor characteristics*					
Histological type	Adenocarcinoma	Ref			
	Squamous carcinoma	1.71	0.82	3.58	0.151
	Others	0.93	0.23	3.81	0.917
Pathological stage	I	Ref			
	II	1.84	0.83	4.09	0.136
	III	1.75	0.73	4.23	0.213
Side	Left	Ref			
	Right	1.66	0.82	3.33	0.157
Lobe	Left	Ref			
	Right upper	0.54	0.19	1.54	0.247
	Right lower	4.14	2.02	8.49	** < 0.0001**
	Other	0.44	0.06	3.46	0.434

Bold letters indicate *p* < 0.05. The minimally adjusted models were adjusted for age and sex

*ECOG* Eastern cooperative oncology group, *FEV 1.0%* forced expiratory volume in 1 s %, *%VC* = % vital capacity, *SHR* subdistribution hazard ratio.

Lobectomy, including bilobectomy, was performed in 798 (81.5%) patients, segmentectomy in 146 (14.9%), and partial resection in 35 (3.6%). Focusing on the surgical procedure, unilateral UPF occurred in 35 (4.4%) of 798 lobectomies, three (2.1%) of 146 segmentectomies, and one (2.8%) of 35 partial resections. Of the 798 lobectomies, including bilobectomies, unilateral UPF occurred in 22 (13.8%) of the 159 right lower lobectomies, eight (4.6%) of the 173 left upper lobectomies, four (1.3%) of the 301 RU lobectomies, and none (0%) of the 103 left lower lobectomies. Moreover, unilateral UPF occurred in four (44.4%) right middle and lower lobectomies (Table 4).

**Table 4:** Summary of operative procedures

	Total	UPF	non-UPF
	(*n* = 979)	(*n* = 39)	(*n* = 940)
*Lobectomy*			
Right upper lobectomy	300	4	296
Right middle lobectomy	59	0	59
Right lower lobectomy	152	19	133
Left upper lobectomy	173	8	165
Left lower lobectomy	103	0	103
*Bilobectomy*			
Right middle and lower lobectomy	9	4	5
Right upper and middle lobectomy	2	0	2
*Segmentectomy*			
Right	63	0	S1 11 S2 10 S3 5 Others 4
Left	83	Upper 2 Lingular 1	Upper 46 Lingular 6 Others 28
*Partial*			
Right	18	0	Upper 13 Middle 1 Lower 3
Left	17	Upper 1	Upper 9 Lower 8

For the subgroup of patients who underwent lobectomy (*n* = 798), Table 5 summarizes the surgical factors and postoperative courses of patients with and without unilateral UPF. VATS was performed in 84.3% of patients who underwent lobectomy. The proportion of patients who underwent VATS was significantly lower in the UPF group than in the non-UPF group. Conversely, a significantly higher proportion of patients underwent chest wall resection or PGA seat placement. Additionally, intraoperative blood loss was significantly higher. Regarding postoperative complications, the proportion of patients with prolonged postoperative drainage (> 5 days) and postoperative pneumonia was significantly higher, and the postoperative stay was significantly longer in the UPF group.

**Table 5:** Characteristics of surgical factors and postoperative course of patients with lobectomy by upper lung field pulmonary fibrosis status. (n = 798)

	Total	UPF	non-UPF	*p*-value
	(*n* = 798)	(*n* = 35)	(*n* = 763)	
*Surgical factors*				
Previous contralateral operation, n (%)	26 (3.3%)	1 (2.9%)	25 (3.3%)	0.89
Surgical approach (VATS), n (%)	673 (84.3%)	23 (65.7%)	650 (85.2%)	**0.002**
Chest wall resection, n (%)	17 (2.1%)	5 (14.3%)	12 (1.6%)	** < 0.001**
Bronchoplasty and/or pulmonary artery plasty, n (%)	33 (4.1%)	3 (8.6%)	30 (3.9%)	0.18
Pleural adhesion, n (%)	167 (20.9%)	8 (22.9%)	159 (20.8%)	0.77
Fibrin glue, n (%)	380 (47.6%)	22 (62.9%)	358 (46.9%)	0.065
PGA sheet, n (%)	162 (20.3%)	12 (34.3%)	150 (19.7%)	**0.035**
Operating time (minutes), mean (SD)	181.6115 (57.70845)	199.9429 (51.30127)	180.7706 (57.87622)	0.055
Blood loss (gram), median (IQR)	107 (50–200)	175 (87–250)	105 (50–200)	**0.014**
*Postoperative complications*				
Chest drainage > 5 days, n (%)	107 (13.4%)	9 (25.7%)	98 (12.8%)	**0.029**
Pleurodesis for air leak, n (%)	39 (4.9%)	2 (5.7%)	37 (4.8%)	0.82
Reoperation for air leak, n (%)	12 (1.5%)	1 (2.9%)	11 (1.4%)	0.50
Pneumonia, n (%)	24 (3.0%)	5 (14.3%)	19 (2.5%)	** < 0.001**
Atelectasis, n (%)	57 (7.1%)	2 (5.7%)	55 (7.2%)	0.74
Pleural effusion, n (%)	35 (4.4%)	2 (5.7%)	33 (4.3%)	0.69
Pleuritis, n (%)	13 (1.6%)	1 (2.9%)	12 (1.6%)	0.56
Hematoma, n (%)	12 (1.5%)	0 (0.0%)	12 (1.6%)	0.45
Atrial fibrillation, n (%)	30 (3.8%)	1 (2.9%)	29 (3.8%)	0.77
Heart failure, n (%)	5 (0.6%)	0 (0.0%)	5 (0.7%)	0.63
Cerebral infarction, n (%)	5 (0.6%)	1 (2.9%)	4 (0.5%)	0.087
Home oxygen therapy, n (%)	9 (1.1%)	1 (2.9%)	8 (1.0%)	0.32
Duration of postoperative stay (days), median (IQR)	7 (6–9)	9 (7–11)	7 (6–8)	** < 0.001**

Bold letters indicate *p* < 0.05

*IQR* interquartile range, *SD* standard deviation, *UPF* upper lung field pulmonary fibrosis, *VATS* video-assisted thoracoscopic surgery

## Comments

Postoperative pulmonary complications after major thoracic surgery, such as pneumonia, atelectasis, and prolonged air leakage, are common and increase hospital mortality, intensive therapy unit admission, and length of hospital stay [14]. Patients developing postoperative pulmonary complications also have worse long-term outcomes; after thoracotomy and lung resection, the mean overall survival was reduced by six months (14). During long-term follow-up after pulmonary resection for lung cancer, slowly progressive lung destruction by fibrosis is a critical issue, and it was recently reported that “unilateral UPF” is a late complication after lung cancer surgery. Tanaka et al. have reported this complication as a unilateral fibrobullous change with an incidence of 3% after lobectomy [5]. In a study by Inafuku et al., the authors referred to unilateral UPF as"unilateral upper lung field pulmonary fibrosis (upper-PF)"and"unilateral PPFE"and reported that the incidence of unilateral UPF development was 4.3% after lung cancer surgery, and the cumulative incidence gradually increased after lung cancer surgery, with a 10-year cumulative incidence of 5.3% [8]. In this study, the incidence of unilateral UPF was 4.0% among all patients after lung cancer surgery, and the 10-year cumulative incidence of unilateral UPF was 5.4%. Furthermore, 76.9% of the unilateral UPF cases presented with severe clinical complications, including respiratory distress, worsening of performance status, progressive body weight loss, and pneumonia. The median interval from lung cancer surgery to the diagnosis of unilateral UPF was 25.5 months (IQR 12.9–45.3 months). Therefore, unilateral UPF should be recognized as a significant late complication associated with lung cancer resection.

The prognosis after the diagnosis of unilateral UPF is reported to be poor, similar to that of PPFE, with a median survival time of 49.3 months, and all causes of death are respiratory diseases [7]. The study included a small number of cases involving diseases other than lung cancer. However, in this study, the 1-year, 3-year, 5-year, and 8-year overall survival rates were favorable at 100%, 97.2%, 87.3%, and 72.1%, respectively. No cases of pulmonary mycosis were identified in this study, which may explain the difference in outcomes.

The mechanism of unilateral UPF is unclear, although it is thought to involve inflammation of the thoracic cavity and/or a postoperative discrepancy between the residual lung and thoracic space. Specifically, a larger resected lung volume may lead to a larger residual thoracic space, which may induce mechanical traction on the residual lung tissue. In this study, UPF occurred most frequently after right lower lobectomy. The right lower lobe occupies 50% of the right thoracic cavity, and it is thought that the postoperative intrathoracic negative pressure in right lower lobectomy is higher than that in other lobectomies. In addition, compared with left lower lobectomy, the remaining upper and middle lobes are strongly pulled caudally due to the absence of a heart. This mechanical traction may contribute to the development of fibrosis, particularly in the upper lung field. Sekine et al. reported an autopsy case of a unilateral UPF that showed evidence of chronic pleuritis [7]. The pulmonary apical cap, reported to be potentially caused by ischemia in the upper lobes and low-grade inflammation in the lung parenchyma [10], has also been reported as a risk factor for unilateral UPF [18] and was a significant risk factor in the present study. In this study, a history of pre- and postoperative pneumonia-related complications was suggested as a possible risk factor for unilateral UPF, and the proportion of PGA sheet use was significantly higher in the UPF group in the subgroup of patients who underwent lobectomy. PGA sheets trigger inflammation in the visceral pleura, leading to the formation of strong and extensive pleural adhesions [19]. Furthermore, pathological findings of resected lung specimens from patients with unilateral UPF showed that 25.6% had interstitial pneumonia and 43.7% had inflammatory changes, despite no evidence of interstitial pneumonia on a preoperative CT scan. These findings suggest that unilateral UPF may be caused by inflammation of the lungs or thoracic cavity, as well as mechanical factors related to the postoperative thoracic space.

In this study, all patients who later developed unilateral UPF initially showed pleural spiculae, indentations, and mediastinal shifts. Unilateral UPF occurred in only one patient (2.9%) after partial resection and in only three patients (2.1%) after segmentectomy; however, it occurred in 13.8% of right lower lobectomies and 44.4% of right middle and lower lobectomies. Moreover, the proportion of patients who underwent chest wall resection was significantly higher in the lobectomy subgroup than in the UPF group. This suggests that chest wall resection may result in a larger volume discrepancy between the thoracic cavity and the residual lung, potentially leading to negative intrathoracic pressure. Right lower lobectomy resulted in a significant volume discrepancy, corresponding to the mismatch between the size of the thoracic cavity and the reduced volume of the residual lung after surgery, as 50% of the right lung volume was resected. These results suggest that negative intrathoracic pressure caused by a discrepancy between the thorax and residual lung may cause substantial distending forces on the pleura, resulting in persistent pleural effusion and chronic pleuritic, pleural spicula, or indentation, especially pleural changes in the apical cap and a mediastinal shift that progresses gradually after surgery.

Recently, the superiority of segmentectomy over lobectomy in the overall survival of patients with peripheral early-stage lung cancer and clinical stage IA non-small cell lung cancer (tumor diameter ≤ 2 cm; consolidation-to-tumor ratio > 0.5) has been reported [20]. In this study, lobectomy was performed in 221 of 337 patients with stage 0 or IA1 disease, including seven in the UPF group who underwent right lower lobectomy. Given that right lower lobectomy removes approximately 50% of the right lung volume, it creates a significant thoracic cavity-lung volume discrepancy, leading to negative intrathoracic pressure and mechanical traction on the pleura. This may explain the higher incidence of persistent pleural effusion (31.2% of 22 right lower lobectomies) and associated complications in these cases. Segmentectomy, with its smaller resection volume, may mitigate these risks and improve long-term outcomes.

This study had some limitations. This retrospective study included a small number of patients with unilateral UPF and lacked a prospective randomized procedure and pathological evaluation of unilateral UPF lesions. Further investigations are required to clarify the clinical and radiological characteristics, mechanisms, and perioperative risk factors of unilateral UPF. Additionally, 16.4% of the patients were followed up for one year, which may have led to an underestimation of the cumulative incidence of unilateral UPF.

## Conclusion

Unilateral UPF is a significant late complication of lung cancer surgery, with an incidence of 4.0% and a 10-year cumulative incidence of 5.4%. Possible risk factors for unilateral UPF include age > 75 years, male sex, low BMI (< 20 kg/m^2^), ischemic heart disease, and a history of pneumonia, emphysema, pulmonary apical cap, and RL tumors. Unilateral UPF should be recognized as a late complication of lung cancer surgery and should be carefully followed over a long period of time in patients with risk factors. 

## Data Availability

Data supporting the findings of this study are available from the corresponding author upon reasonable request.
